# Supplementary material for: A prolonged stress rat model recapitulates some PTSD-like changes in sleep and neuronal connectivity
Source: Commun Biol. 2023 Jul 12;6:716. doi: 10.1038/s42003-023-05090-9 (PMC10338557; doi:10.1038/s42003-023-05090-9)
Supplement: Supplementary file 2 — Supplementary Information [file 42003_2023_5090_MOESM2_ESM.pdf]

SUPPLEMENTARY INFORMATION

A prolonged stress rat model recapitulates some PTSD-like changes in sleep and neuronal connectivity

Yun Lo[1#], Pei-Lu Yi[2*], Yi-Tse Hsiao[1], Tung-Yen Lee[3], Fang-Chia Chang[1,3,4,5,6*]

Author information

<u>Authors and Affiliations</u>

[1] **Department of Veterinary Medicine, School of Veterinary Medicine, National Taiwan University, Taipei, 10617, Taiwan**

[2] **Department of Sport Management, College of Tourism, Leisure and Sports, Aletheia University, New Taipei City, 25103, Taiwan**

[3] **Graduate Institute of Brain & Mind Sciences, College of Medicine, National Taiwan University, Taipei, 110225, Taiwan**

[4] **Neurobiology & Cognitive Science Center, National Taiwan University, Taipei, 10617, Taiwan**

[5] **Graduate Institute of Acupuncture Science, College of Chinese Medicine, China Medical University, Taichung, 40402, Taiwan**

[6] **Department of Medicine, College of Medicine, China Medical University, Taichung, 40402, Taiwan**

Corresponding authors

Correspondence to Fang-Chia Chang, Ph.D.[§] or Pei-Lu Yi, Ph.D. [†].

Addresses: [§]School of Veterinary Medicine, National Taiwan University, No. 1, Sec. 4., Roosevelt Road, Taipei, Taiwan, Tel: +886-2-3366-3883, Email: fchang@ntu.edu.tw

[†]Department of Sport Management, Aletheia University, New Taipei City, Taiwan, Email: pyi67@hotmail.com

Supplementary Figures and Legends

Supplementary Fig. 1: Contextual fear memory retrieval enhanced PFC-BLA-vHPC circuitries' theta intensity and coherence.

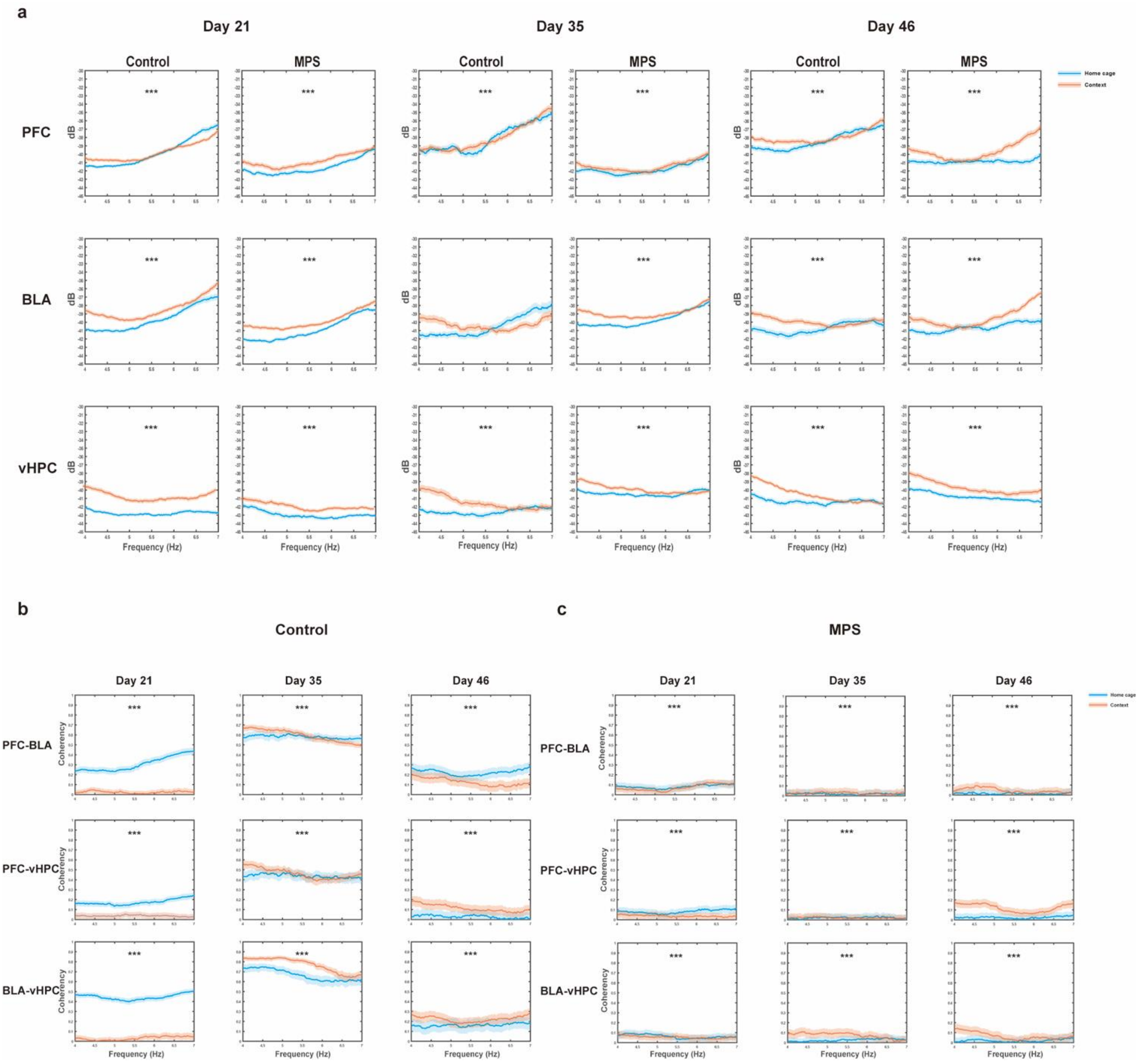

a Theta spectral power alterations post-contextual retrieval. Control n=6, MPS n=6. Paired *t*-test: $^{***}p<0.001$: home cage vs. context retrieval. **b,c** Theta powers coherency within PFC-BLA, PFC-vHPC, and BLA-vHPC circuitries

strengthened post-contextual retrieval in control (**b**) and MPS (**c**) groups. Control n=6, MPS n=6. Paired *t*-test: ***$p<0.001$: home cage vs. context retrieval. Values represent the mean ± SEM in a 4-7 Hz frequency band.

Supplementary Fig. 2: Control and MPS varying theta power and coherence comparison during memory retrievals within PFC-BLA-vHPC circuitry.

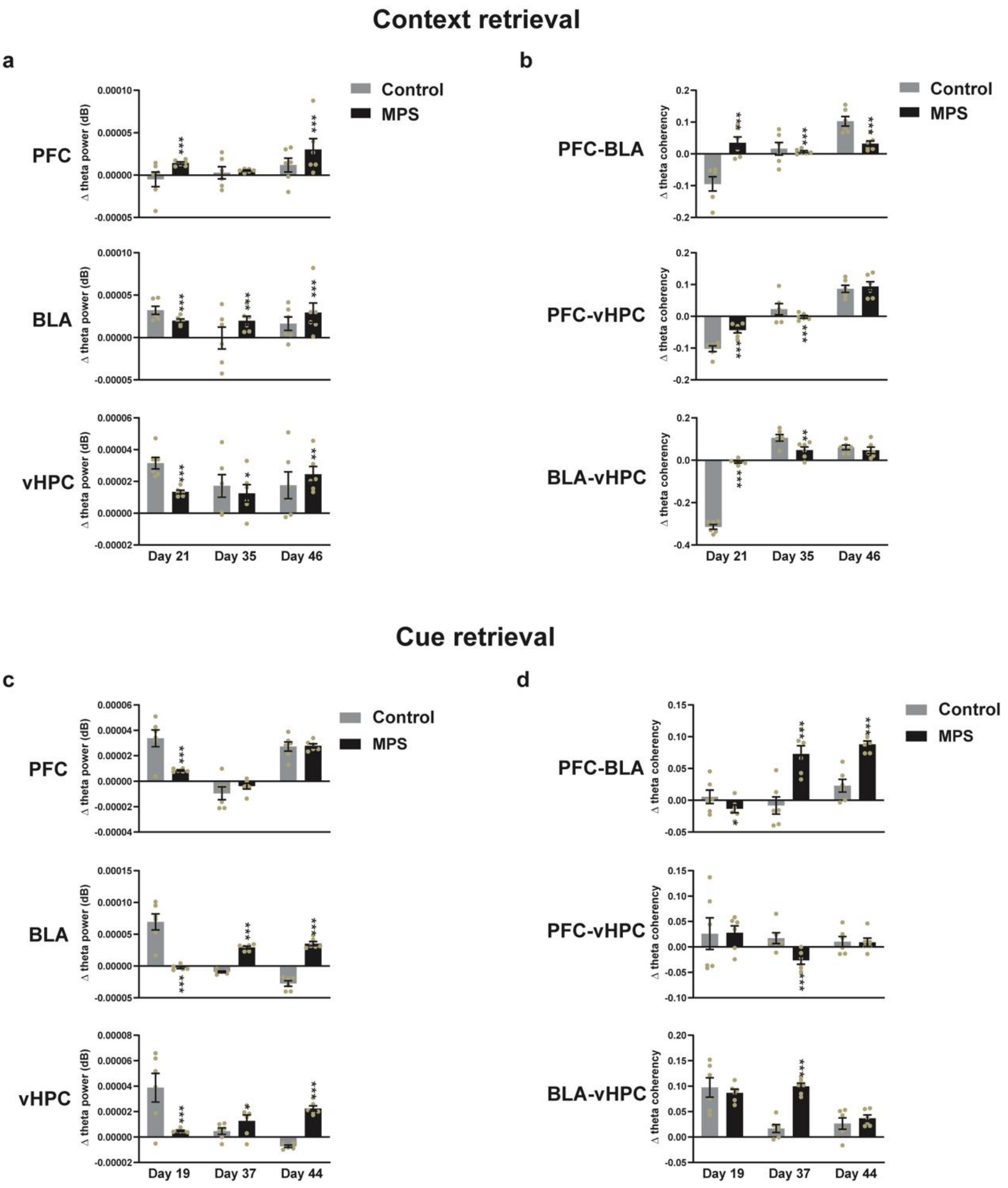

a Theta power variations (context minus home cage) during context retrieval in PFC, BLA, and vHPC regions. Two-way repeated measures ANOVA: time

x group interaction at PFC $F_{(1,10)}$=0.786, p=0.469, at BLA $F_{(1,10)}$= 2.747, p=0.088, at vHPC $F_{(1,10)}$=7.792, p<0.05; main effect of group at PFC $F_{group(1,10)}$=3.982, p=0.074, at BLA $F_{group(1,10)}$=0.716, p=0.417, at vHPC $F_{group(1,10)}$=0.555, p=0.474. Control n=6, MPS n=6. Bonferroni's post hoc comparison: $^{*}p$<0.05, $^{**}p$<0.01, $^{***}p$<0.001: control vs. MPS groups. **b** Theta coherence variations (context minus home cage) within PFC-BLA, PFC-vHPC, and BLA-vHPC circuitries during context retrieval. Two-way repeated measures ANOVA: time x group interaction at PFC-BLA $F_{(1,10)}$=5.982, p<0.05, at PFC-vHPC $F_{(1,10)}$=1.918, p=0.173, at BLA-vHPC $F_{(1,10)}$=17.542, p<0.0001; main effect of group at PFC-BLA $F_{group(1,10)}$=6.871, p<0.05, at PFC-vHPC $F_{group(1,10)}$=5.218, p<0.05, at BLA-vHPC $F_{group(1,10)}$=2.074, p=0.180. Control n=6, MPS n=6. Bonferroni's post hoc comparison: $^{**}p$<0.01, $^{***}p$<0.001: control vs. MPS groups. **c** Theta power variations (1-second post-cue minus 1-second pre-cue) during cue retrieval in PFC, BLA, and vHPC regions. Two-way repeated measures ANOVA: time x group interaction at PFC $F_{(1,10)}$=9.891, p<0.001, at BLA $F_{(1,10)}$=65.196, p<0.0001, at vHPC $F_{(1,10)}$=17.110, p<0.0001; main effect of group at PFC $F_{group(1,10)}$=3.686, p=0.084, at BLA $F_{group(1,10)}$=7.102, p<0.05, at vHPC $F_{group(1,10)}$=0.132, p=0.724. Control n=6, MPS n=6. Bonferroni's post hoc comparison: $^{*}p$<0.05, $^{***}p$<0.001:

control vs. MPS groups. **d** Theta coherence variations (1-second post-cue minus 1-second pre-cue) within PFC-BLA, PFC-vHPC, and BLA-vHPC circuitries during cue retrieval. Two-way repeated measures ANOVA: time x group interaction at PFC-BLA $F_{(1,10)}$=14.058, p<0.0001, at PFC-vHPC $F_{(1,10)}$= 1.289, p=0.297, at BLA-vHPC $F_{(1,10)}$=8.644, p<0.05; main effect of group at PFC-BLA $F_{group(1,10)}$=26.399, p<0.0001, at PFC-vHPC $F_{group(1,10)}$=1.235, p=0.293, at BLA-vHPC $F_{group(1,10)}$=19.182, p<0.001. Control n=6, MPS n=6. Bonferroni's post hoc comparison: $^{*}p$<0.05, $^{***}p$<0.001: control vs. MPS groups. Values represent the mean ± SEM. The detail statistical values were presented in Supplementary Table. 5.

Supplementary Fig. 3: Auditory-cue fear memory retrieval enhanced PFC-BLA-vHPC circuitry theta intensity and coherency.

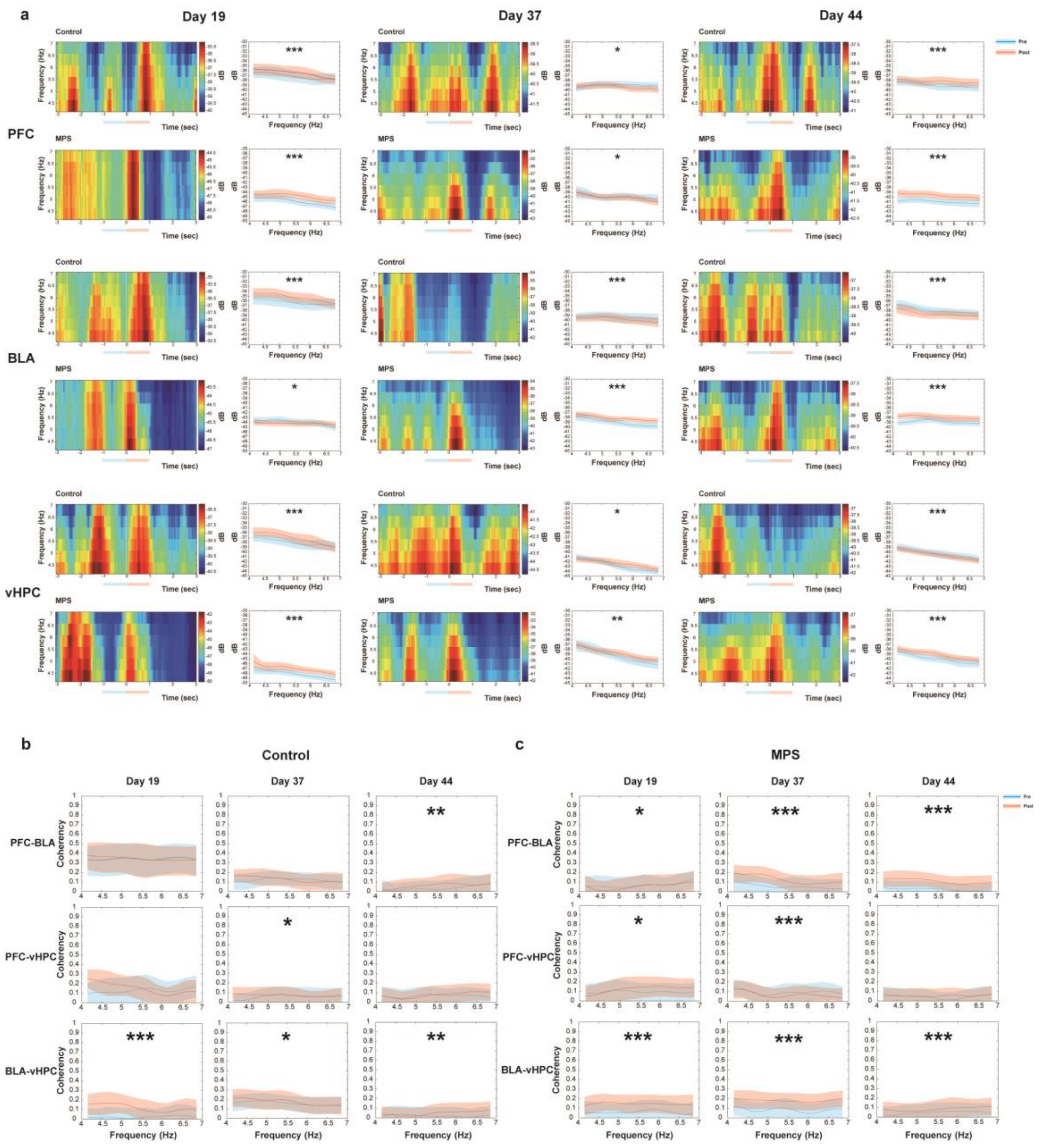

a Theta spectral power alteration post-cue retrieval compared to the baseline acquired from the home cage's pre-cue tone period. Control n=6, MPS n=6. Paired *t*-test: $^{*}p<0.05$, $^{**}p<0.01$, $^{***}p<0.001$: pre-cue tone vs. post-cue tone. **b,c** Theta power coherencies within PFC-BLA, PFC-vHPC, and BLA-vHPC

circuitries strengthened post-cue retrieval in control (**b**) and MPS (**c**) groups. Control n=6, MPS n=6. Paired *t*-test: $^{*}p<0.05$, $^{**}p<0.01$, $^{***}p<0.001$: pre-cue tone vs. post-cue tone. Values represent the mean ± SEM in a 4-7 Hz frequency band.

Supplementary Fig. 4: Fear memory retrieval promoted PFC, BLA, and vHPC theta activity during behavioral tasks.

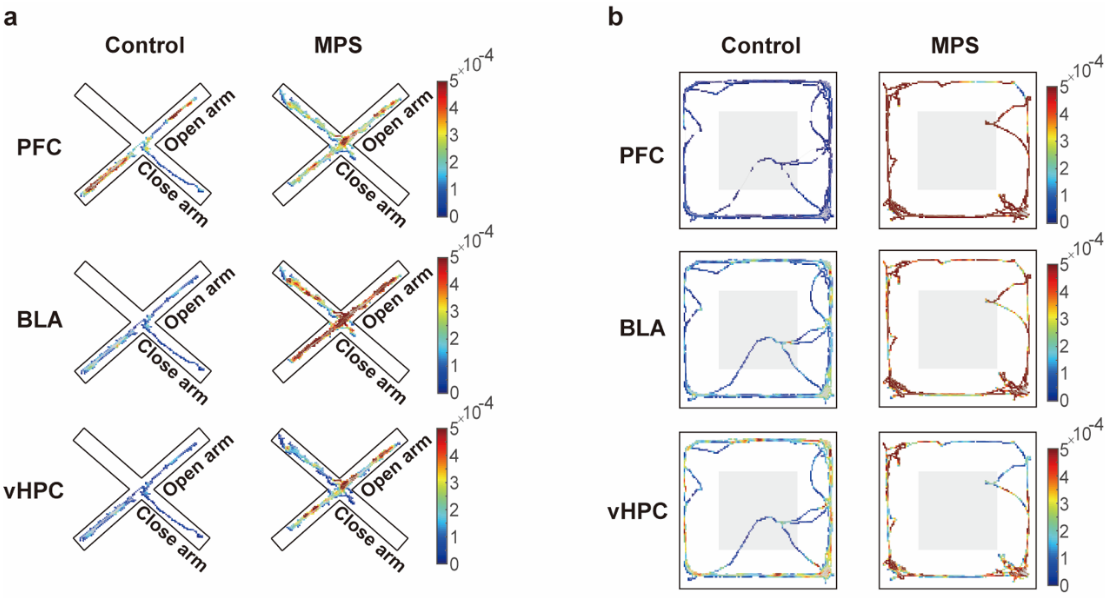

a PFC, BLA, and vHPC theta power spectrograms and rats' Day 44 EPM traveling path were simultaneously plotted. **b** PFC, BLA, and vHPC theta power spectrograms and rats' Day 37 OFT traveling path were simultaneously plotted.

Supplementary Fig. 5: Repeated fear memory retrievals did not influence longitudinal freezing effects post-MPS.

a

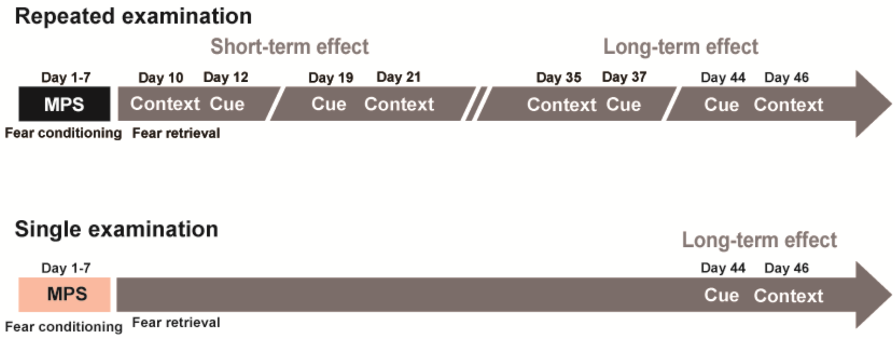

b

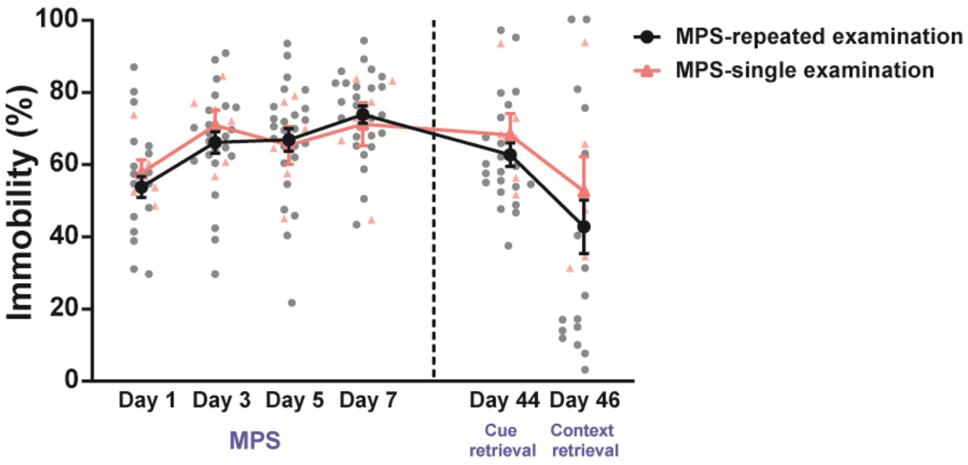

a Repeated applied memory (black box; see “Methods”) and single administrated (pink box) retrieval trails in the MPS group and during the long-term period post-MPS manipulation experimental protocols, respectively. The single retrieval administrated MPS group experienced the same MPS process on Days 1-7 and remained in the home cage without disturbance for six weeks to detect long-term memory recall effects on Days 44 and 46. **b** Repeated and single retrieval examination group average immobility (%) had

no statistical differences during the MPS manipulated period and long-term cue (light-sound compounded cue tone) and contextual retrievals. GEE: Group x Time interaction=3.737, $p<0.001$; B=52.611, 95% CI 35.470-69.752, $p<0.001$. Repeated n=26, single n=6 on Day 1; repeated n=24, single n=6 on Day 3; repeated n=25, single n=6 on Day 5; repeated n=25, single n=6 on Day 7; repeated n=22, single n=6 on Day 44; repeated n=25, single n=6 on Day 46. Values represent the mean ± SEM.

Supplementary Fig. 6: Stressful MPS effects were extensively more substantial than SPS.

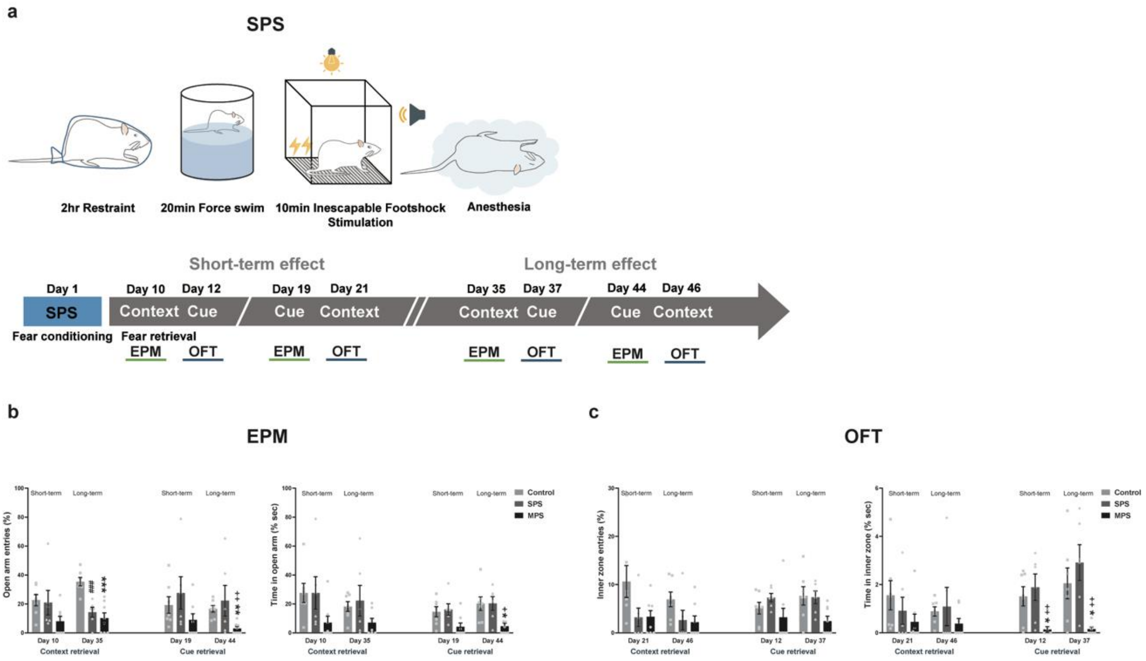

a SPS (blue box) with fear conditioning, fear memory retrieval, and behavior tasks (EPM with green line; OFT with blue line) were performed within 50 days (see "Methods"). **b** Behavioral alterations during the 5-minute EPM task. SPS group's open arm entry (%) declined after long-term context retrieval (Day 35). Open arm two-way repeated measures ANOVA: time x group interaction $F_{(1,18)}$=3.329, p=0.063; main effect of group $F_{group(1,18)}$=8.342, p<0.01 in context recall entry frequency (%) (left). Time x group interaction $F_{(1,18)}$=0.147, p=0.864; main effect of group $F_{group(1,18)}$=7.052, p<0.01 in cue recall entry frequency (%) (left). Time x group interaction $F_{(1,18)}$=0.272, p=0.765; main effect of group $F_{group(1,18)}$=11.522, p<0.001 in cue recall

accumulated time (%) (right). Control n=7, SPS n=6, MPS n=8. Bonferroni's post hoc comparison: $^{*}p<0.05$, $^{**}p<0.01$, $^{***}p<0.001$: control vs. MPS; $^{\#\#\#}p<0.001$: control vs. SPS; $^{+}p<0.05$, $^{++}p<0.01$: SPS vs. MPS. **c** Behavioral alterations during 10-minute OFT. SPS group's inner zone entry (%) declined after short-term context retrieval (Day 21). Inner zone two-way repeated measures ANOVA: time x group interaction $F_{(1,18)}=0.612$, $p=0.553$; main effect of group $F_{group(1,18)}=14.076$, $p<0.0001$ in cue recall accumulated time (%) (right). Control n=7, SPS n=6, MPS n=8. Bonferroni's post hoc comparison: $^{*}p<0.05$: control vs. MPS; $^{++}p<0.01$: SPS vs. MPS. Values represent the mean ± SEM. The detail statistical values were presented in Supplementary Table. 6.

Supplementary Fig. 7: Sleep-wake activity alterations after the MPS and SPS manipulations.

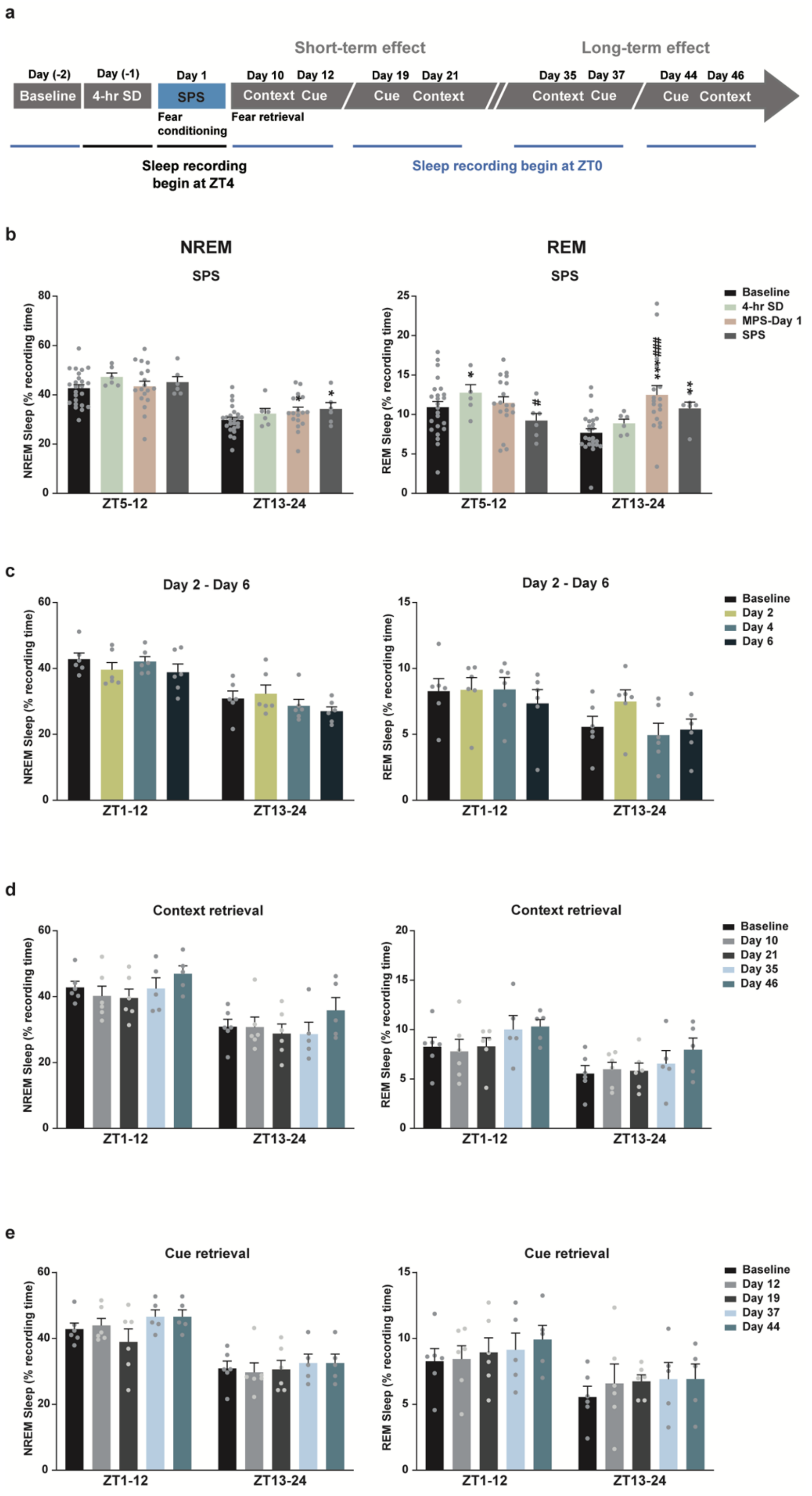

a Sleep-wake activity recording lasted seven weeks. Sleep-wake activity recording began at ZT4 and continued for 20 hours during the 4-hr SD (Day (-1)) and SPS day (**b**), and from ZT0 for 24 hours during baseline recording day (Day (-2)), Days 2-6, and after fear memory retrievals (**c-e**) (see "Methods"). **b** Average SPS-altered NREM (left) and REM (right) sleep time (%) during light and dark periods. SPS manipulation during ZT1-4 primarily decreased REM sleep for 8 hours (ZT5-12) and compensatively increased NREM and REM sleep during the following dark period (ZT13-24). Baseline n=24, 4-hr SD n=6, MPS-Day 1 n=18, SPS n=6. One-way ANOVA: $F_{(3,644)}$=3.604, p<0.01 for ZT13-24 NREM sleep; $F_{(3,428)}$=3.343, p<0.05 and $F_{(3,644)}$=22.174, p<0.0001 for ZT5-12 and ZT13-24 REM sleep, respectively. Baseline n=24, 4-hr SD n=6, MPS-Day 1 n=18, SPS n=6. Tukey's multiple comparison: $^{*}p$<0.05, $^{**}p$<0.01, $^{***}p$<0.001: baseline vs. 4-hr SD, MPS-Day 1, and SPS, respectively; $^{\#}p$<0.05, $^{\#\#\#}p$<0.001: 4-hr SD vs. MPS-Day 1 and SPS, respectively. **c** Average NREM (left) and REM (right) sleep time (%) on Days 2, 4, and 6 did not change after SPS manipulation. Baseline and Days 2-6 n=6. **d, e** Average NREM (left) and REM (right) sleep time (%) after context retrieval (**d**) and cue retrieval (**e**). Both contextual and cue retrievals did not affect the amount of sleep during

the following 24 hours. Baseline n=6, Days 10, 12, 19, and 21 n=6, Days 35, 37, 44, and 46 n=5. Values represent the mean ± SEM.

Supplementary Fig. 8: SPS and fear memory retrieval facilitated SWAs, REM sleep, and waking theta power.

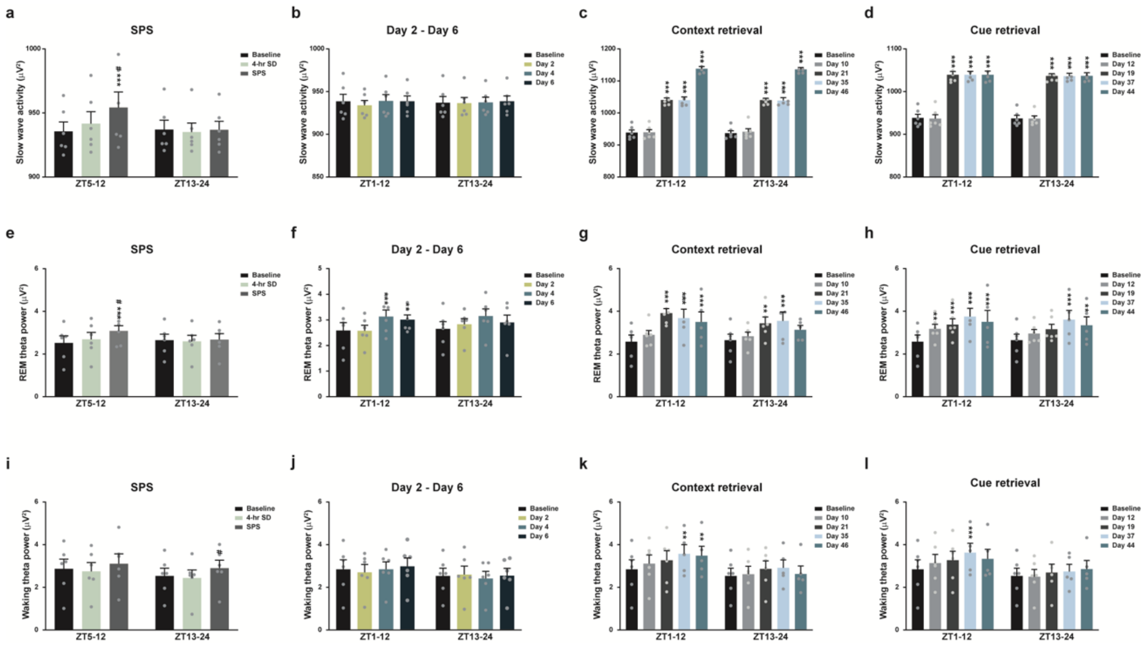

a-d Average SWAs during NREM sleep light and dark periods. SWAs were enhanced after SPS (**a**), short- (**c**), and long-term (**d**) fear memory retrievals. One-way ANOVA: $F_{(2,141)}$=6.940, p=0.001 during ZT5-12 in (**a**); $F_{(4,330)}$=1228.162, p<0.001 and $F_{(4,317)}$=1127.574, p<0.0001 during ZT1-12 and ZT13-24 in (**c**); $F_{(4,319)}$=483.193, p<0.0001 and $F_{(4,318)}$=770.255, p<0.0001 during ZT1-12 and ZT13-24 in (**d**). Tukey's multiple comparison: $^{***}p$<0.001: baseline vs. 4-hr SD and SPS, and baseline vs. fear memory retrieval days; $^{\#}p$<0.05: 4-hr SD vs. SPS. **e-h** Average REM sleep theta powers were collected after SPS protocol (**e**), Days 2-6 after SPS manipulation (**f**), context retrieval (**g**), and cue retrieval (**h**). The SPS protocol increased theta intensity

during REM sleep (**e**) and during post-treated Days 4 and 6 (**f**). REM sleep theta powers were enhanced during context (**g**) and cue retrieval (**h**). One-way ANOVA: $F_{(2,134)}=4.729$, $p<0.01$ during ZT5-12 in (**e**); $F_{(3,264)}=7.979$, $p<0.0001$ during ZT1-12 in (**f**); $F_{(4,322)}=18.011$, $p<0.0001$ and $F_{(4,280)}=6.952$, $p<0.0001$ during ZT1-12 and ZT13-24 in (**g**); $F_{(4,324)}=13.659$, $p<0.0001$ and $F_{(4,272)}=9.031$, $p<0.0001$ during ZT1-12 and ZT13-24 in (**h**). Tukey's multiple comparison: $^{**}p<0.01$, $^{***}p<0.001$: baseline vs. 4-hr SD and SPS, baseline vs. Days 2-6, and baseline vs. fear memory retrieval days; $^{\#}p<0.05$: 4-hr SD vs. SPS. **i-l** Average theta powers during wakefulness after SPS (**i**), Days 2-6 after SPS manipulation (**j**), and fear memory retrievals (**k, l**). Waking theta power was elevated after SPS protocol (**i**). Both context (**k**) and cue retrieval (**l**) increased theta power intensities during the long-term period. One-way ANOVA: $F_{(2,213)}=4.383$, $p<0.05$ during ZT13-24 in (**i**); $F_{(4,331)}=4.672$, $p<0.001$ during ZT1-12 in (**k**); $F_{(4,331)}=4.400$, $p<0.01$ during ZT1-12 in (**l**). Tukey's multiple comparison: $^{**}p<0.01$, $^{***}p<0.001$: baseline vs. fear memory retrieval days; $^{\#}p<0.05$: 4-hr SD vs. SPS. Baseline n=6, 4-hr SD n=6, SPS n=6 in (**a**), (**e**), and (**i**); baseline and Days 2-6 n=6 in (**b**), (**f**), and (**j**); baseline n=6, Days 10, 12, 19, and 21 n=6, Days 35, 37, 44, and 46 n=5. in (**c**), (**d**), (**g**), (**h**), (**k**), and (**l**). Values represent the mean ± SEM.

Supplementary Tables and Legends

Supplementary Table. 1: Statistical results of Fig. 2.

Context retrieval				Cue retrieval			
PFC				PFC			
Days/ Effect	Group x Environment	Main effect of Group	Main effect of Environment	Days/ Effect	Group x Period	Main effect of Group	Main effect of Period
Day 21	$F_{(1,10)}=4.802$, $p=0.053$	$F_{(1,10)}=3.500$, $p=0.091$	$F_{(1,10)}=1.101$, $p=0.319$	Day 19	$F_{(1,10)}=14.200$, $p<0.01$	$F_{(1,10)}=182.311$, $p<0.0001$	$F_{(1,10)}=39.646$, $p<0.0001$
Day 35	$F_{(1,10)}=0.129$, $p=0.727$	$F_{(1,10)}=11.223$, $p<0.05$	$F_{(1,10)}=1.252$, $p=0.289$	Day 37	$F_{(1,10)}=1.083$, $p=0.323$	$F_{(1,10)}=13.954$, $p<0.05$	$F_{(1,10)}=5.633$, $p<0.05$
Day 46	$F_{(1,10)}=1.527$, $p=0.245$	$F_{(1,10)}=13.795$, $p<0.01$	$F_{(1,10)}=7.804$, $p<0.05$	Day 44	$F_{(1,10)}=0.032$, $p=0.862$	$F_{(1,10)}=70.602$, $p<0.0001$	$F_{(1,10)}=185.645$, $p<0.0001$
BLA				BLA			
Days/ Effect	Group x Environment	Main effect of Group	Main effect of Environment	Days/ Effect	Group x Period	Main effect of Group	Main effect of Period
Day 21	$F_{(1,10)}=5.549$, $p<0.05$	$F_{(1,10)}=2.597$, $p=0.138$	$F_{(1,10)}=100.749$, $p<0.0001$	Day 19	$F_{(1,10)}=31.945$, $p<0.0001$	$F_{(1,10)}=193.934$, $p<0.0001$	$F_{(1,10)}=27.794$, $p<0.0001$
Day 35	$F_{(1,10)}=2.064$, $p=0.181$	$F_{(1,10)}=4.948$, $p<0.05$	$F_{(1,10)}=1.843$, $p=0.204$	Day 37	$F_{(1,10)}=215.244$, $p<0.0001$	$F_{(1,10)}=13.337$, $p<0.01$	$F_{(1,10)}=58.858$, $p<0.0001$
Day 46	$F_{(1,10)}=0.887$, $p=0.368$	$F_{(1,10)}=0.715$, $p=0.418$	$F_{(1,10)}=10.984$, $p<0.01$	Day 44	$F_{(1,10)}=139.108$, $p<0.0001$	$F_{(1,10)}=0.450$, $p=0.517$	$F_{(1,10)}=1.733$, $p=0.217$
vHPC				vHPC			
Days/ Effect	Group x Environment	Main effect of Group	Main effect of Environment	Days/ Effect	Group x Period	Main effect of Group	Main effect of Period
Day 21	$F_{(1,10)}=22.761$, $p<0.001$	$F_{(1,10)}=9.769$, $p<0.05$	$F_{(1,10)}=139.202$, $p<0.0001$	Day 19	$F_{(1,10)}=9.034$, $p<0.05$	$F_{(1,10)}=104.841$, $p<0.0001$	$F_{(1,10)}=15.024$, $p<0.01$
Day 35	$F_{(1,10)}=0.266$, $p=0.617$	$F_{(1,10)}=56.217$, $p<0.0001$	$F_{(1,10)}=10.905$, $p<0.01$	Day 37	$F_{(1,10)}=2.312$, $p=0.159$	$F_{(1,10)}=19.732$, $p<0.001$	$F_{(1,10)}=10.530$, $p<0.01$
Day 46	$F_{(1,10)}=0.514$, $p=0.490$	$F_{(1,10)}=2.957$, $p=0.116$	$F_{(1,10)}=18.615$, $p<0.01$	Day 44	$F_{(1,10)}=172.006$, $p<0.0001$	$F_{(1,10)}=2.561$, $p=0.141$	$F_{(1,10)}=44.881$, $p<0.0001$

The *F*-statistic and *p*-value of two-way repeated measures ANOVA factors interaction (group x environment/period) and main effects (group and environment/period) in each brain region. The corresponding main effects after Bonferroni’s post hoc comparison with significant differences displayed in Fig. 2 were highlighted with the gray background.

Supplementary Table. 2: Statistical results of Fig. 3.

Context retrieval				Cue retrieval			
PFC-BLA				PFC-BLA			
Days/ Effect	Group x Environment	Main effect of Group	Main effect of Environment	Days/ Effect	Group x Period	Main effect of Group	Main effect of Period
Day 21	$F_{(1,10)}$=19.826, p<0.001	$F_{(1,10)}$=101.392, p<0.0001	$F_{(1,10)}$=4.170, p=0.068	Day 19	$F_{(1,10)}$=2.373, p=0.154	$F_{(1,10)}$=598.203, p<0.0001	$F_{(1,10)}$=0.437, p=0.524
Day 35	$F_{(1,10)}$=0.223, p=0.647	$F_{(1,10)}$=1313.358, p<0.0001	$F_{(1,10)}$=1.351, p=0.272	Day 37	$F_{(1,10)}$=19.142, p<0.001	$F_{(1,10)}$=2.446, p=0.149	$F_{(1,10)}$=12.141, p<0.01
Day 46	$F_{(1,10)}$=16.131, p<0.01	$F_{(1,10)}$=22.104, p<0.001	$F_{(1,10)}$=59.034, p<0.0001	Day 44	$F_{(1,10)}$=34.364, p<0.0001	$F_{(1,10)}$=1.627, p=0.231	$F_{(1,10)}$=99.491, p<0.0001
PFC-vHPC				PFC-vHPC			
Days/ Effect	Group x Environment	Main effect of Group	Main effect of Environment	Days/ Effect	Group x Period	Main effect of Group	Main effect of Period
Day 21	$F_{(1,10)}$=21.211, p<0.001	$F_{(1,10)}$=66.890, p<0.0001	$F_{(1,10)}$=131.485, p<0.0001	Day 19	$F_{(1,10)}$=0.002, p=0.962	$F_{(1,10)}$=6.428, p<0.05	$F_{(1,10)}$=2.497, p=0.145
Day 35	$F_{(1,10)}$=1.786, p=0.211	$F_{(1,10)}$=968.437, p<0.0001	$F_{(1,10)}$=131.485, p<0.0001	Day 37	$F_{(1,10)}$=11.122, p<0.01	$F_{(1,10)}$=3.168, p=0.105	$F_{(1,10)}$=0.510, p=0.492
Day 46	$F_{(1,10)}$=0.182, p=0.679	$F_{(1,10)}$=0.002, p=0.961	$F_{(1,10)}$=94.912, p<0.0001	Day 44	$F_{(1,10)}$=0.008, p=0.929	$F_{(1,10)}$=5.245, p<0.05	$F_{(1,10)}$=2.265, p=0.163
BLA-vHPC				BLA-vHPC			
Days/ Effect	Group x Environment	Main effect of Group	Main effect of Environment	Days/ Effect	Group x Period	Main effect of Group	Main effect of Period
Day 21	$F_{(1,10)}$=596.370, p<0.0001	$F_{(1,10)}$=1353.968, p<0.0001	$F_{(1,10)}$=662.074, p<0.0001	Day 19	$F_{(1,10)}$=0.268, p=0.616	$F_{(1,10)}$=14.535, p<0.01	$F_{(1,10)}$=83.048, p<0.0001
Day 35	$F_{(1,10)}$=7.200, p<0.05	$F_{(1,10)}$=620.400, p<0.0001	$F_{(1,10)}$=50.214, p<0.0001	Day 37	$F_{(1,10)}$=69.187, p<0.0001	$F_{(1,10)}$=8.161, p<0.05	$F_{(1,10)}$=136.010, p<0.0001
Day 46	$F_{(1,10)}$=0.491, p=0.500	$F_{(1,10)}$=216.516, p<0.0001	$F_{(1,10)}$=33.200, p<0.0001	Day 44	$F_{(1,10)}$=0.633, p=0.445	$F_{(1,10)}$=17.805, p<0.01	$F_{(1,10)}$=24.256, p<0.001

The *F*-statistic and *p*-value of two-way repeated measures ANOVA factors interaction (group x environment/period) and main effects (group and environment/period) in each coherence area. The corresponding main effects after Bonferroni's post hoc comparison with significant differences displayed in Fig. 3 were highlighted with the gray background.

Supplementary Table. 3: Statistical results of Fig. 4.

Context retrieval			
**Control**			
PFC-BLA			
Days/ Effect	Environment x Direction	Main effect of Direction	Main effect of Environment
Day 21	$F_{(1,5)}$=2.469, p=0.177	$F_{(1,5)}$=2.505, p=0.174	$F_{(1,5)}$=1.798, p=0.238
Day 35	$F_{(1,5)}$=0.282, p=0.168	$F_{(1,5)}$=0.268, p=0.627	$F_{(1,5)}$=12.612, p<0.005
Day 46	$F_{(1,5)}$=2.380, p=0.184	$F_{(1,5)}$=2.359, p=0.185	$F_{(1,5)}$=2.450, p=0.178
PFC-vHPC			
Days/ Effect	Environment x Direction	Main effect of Direction	Main effect of Environment
Day 21	$F_{(1,5)}$=2.095, p=0.207	$F_{(1,5)}$=2.256, p=0.193	$F_{(1,5)}$=1.870, p=0.230
Day 35	$F_{(1,5)}$=4.319, p=0.092	$F_{(1,5)}$=7.970, p<0.05	$F_{(1,5)}$=26.073, p<0.01
Day 46	$F_{(1,5)}$=2.256, p=0.193	$F_{(1,5)}$=2.262, p=0.193	$F_{(1,5)}$=1.965, p=0.220
BLA-vHPC			
Days/ Effect	Environment x Direction	Main effect of Direction	Main effect of Environment
Day 21	$F_{(1,5)}$=0.033, p=0.863	$F_{(1,5)}$=26.652, p<0.01	$F_{(1,5)}$=1.888, p=0.228
Day 35	$F_{(1,5)}$=3.052, p=0.141	$F_{(1,5)}$=0.543, p=0.494	$F_{(1,5)}$=0.054, p=0.825
Day 46	$F_{(1,5)}$=1.135, p=0.335	$F_{(1,5)}$=1.743, p=0.244	$F_{(1,5)}$=4.676, p=0.083
**MPS**			
PFC-BLA			
Days/ Effect	Environment x Direction	Main effect of Direction	Main effect of Environment
Day 21	$F_{(1,5)}$=1.134, p=0.336	$F_{(1,5)}$=11.044, p<0.05	$F_{(1,5)}$=3.613, p=0.116
Day 35	$F_{(1,5)}$=0.092, p=0.774	$F_{(1,5)}$=0.530, p=0.449	$F_{(1,5)}$=0.341, p=0.584
Day 46	$F_{(1,5)}$=1.268, p=0.311	$F_{(1,5)}$=1.079, p=0.346	$F_{(1,5)}$=0.814, p=0.408
PFC-vHPC			
Days/ Effect	Environment x Direction	Main effect of Direction	Main effect of Environment
Day 21	$F_{(1,5)}$=0.321, p=0.596	$F_{(1,5)}$=5.039, p=0.075	$F_{(1,5)}$=0.127, p=0.736
Day 35	$F_{(1,5)}$=2.670, p=0.163	$F_{(1,5)}$=2.281, p=0.191	$F_{(1,5)}$=0.587, p=0.478
Day 46	$F_{(1,5)}$=0.265, p=0.629	$F_{(1,5)}$=0.217, p=0.661	$F_{(1,5)}$=7.203, p<0.05
BLA-vHPC			
Days/ Effect	Environment x Direction	Main effect of Direction	Main effect of Environment
Day 21	$F_{(1,5)}$=2.964, p=0.146	$F_{(1,5)}$=0.325, p=0.593	$F_{(1,5)}$=5.094, p=0.074
Day 35	$F_{(1,5)}$=1.349, p=0.298	$F_{(1,5)}$=1.223, p=0.1319	$F_{(1,5)}$=2.274, p=0.192
Day 46	$F_{(1,5)}$=0.959, p=0.372	$F_{(1,5)}$=0.957, p=0.373	$F_{(1,5)}$=0.955, p=0.373

Cue retrieval			
**Control**			
PFC-BLA			
Days/ Effect	Period x Direction	Main effect of Direction	Main effect of Period
Day 19	$F_{(1,5)}$=0.257, p=0.634	$F_{(1,5)}$=0.691, p=0.444	$F_{(1,5)}$=1.053, p=0.352
Day 37	$F_{(1,5)}$=0.113, p=0.751	$F_{(1,5)}$=57.217, p<0.001	$F_{(1,5)}$=0.583, p=0.480
Day 44	$F_{(1,5)}$=0.047, p=0.836	$F_{(1,5)}$=6.579, p<0.05	$F_{(1,5)}$=1.241, p=0.316
PFC-vHPC			
Days/ Effect	Period x Direction	Main effect of Direction	Main effect of Period
Day 19	$F_{(1,5)}$=3.900, p=0.105	$F_{(1,5)}$=1.796, p=0.238	$F_{(1,5)}$=0.374, p=0.568
Day 37	$F_{(1,5)}$=0.042, p=0.846	$F_{(1,5)}$=14.736, p<0.05	$F_{(1,5)}$=5.635, p=0.064
Day 44	$F_{(1,5)}$=0.906, p=0.385	$F_{(1,5)}$=0.421, p=0.545	$F_{(1,5)}$=1.556, p=0.267
BLA-vHPC			
Days/ Effect	Period x Direction	Main effect of Direction	Main effect of Period
Day 19	$F_{(1,5)}$=0.830, p=0.404	$F_{(1,5)}$=29.845, p<0.01	$F_{(1,5)}$=0.880, p=0.391
Day 37	$F_{(1,5)}$=0.169, p=0.698	$F_{(1,5)}$=4.128, p=0.098	$F_{(1,5)}$=2.227, p=0.196
Day 44	$F_{(1,5)}$=0.185, p=0.685	$F_{(1,5)}$=4.957, p=0.077	$F_{(1,5)}$=2.541, p=0.172
**MPS**			
PFC-BLA			
Days/ Effect	Period x Direction	Main effect of Direction	Main effect of Period
Day 19	$F_{(1,5)}$=0.609, p=0.470	$F_{(1,5)}$=0.025, p=0.881	$F_{(1,5)}$=0.185, p=0.685
Day 37	$F_{(1,5)}$=32.303, p<0.01	$F_{(1,5)}$=2.021, p=0.214	$F_{(1,5)}$=1.102, p=0.342
Day 44	$F_{(1,5)}$=4.255, p=0.094	$F_{(1,5)}$=21.055, p<0.01	$F_{(1,5)}$=0.286, p=0.616
PFC-vHPC			
Days/ Effect	Period x Direction	Main effect of Direction	Main effect of Period
Day 19	$F_{(1,5)}$=1.406, p=0.289	$F_{(1,5)}$=2.660, p=0.164	$F_{(1,5)}$=5.505, p=0.066
Day 37	$F_{(1,5)}$=0.587, p=0.263	$F_{(1,5)}$=29.987, p<0.01	$F_{(1,5)}$=0.156, p=0.709
Day 44	$F_{(1,5)}$=2.785, p=0.156	$F_{(1,5)}$=18.820, p<0.01	$F_{(1,5)}$=3.501, p=0.120
BLA-vHPC			
Days/ Effect	Period x Direction	Main effect of Direction	Main effect of Period
Day 19	$F_{(1,5)}$=4.282, p=0.093	$F_{(1,5)}$=3.125, p=0.137	$F_{(1,5)}$=7.132, p<0.05
Day 37	$F_{(1,5)}$=1.661, p=0.254	$F_{(1,5)}$=20.390, p<0.01	$F_{(1,5)}$=1.215, p=0.320
Day 44	$F_{(1,5)}$=4.877, p=0.078	$F_{(1,5)}$=15.132, p<0.05	$F_{(1,5)}$=0.068, p=0.805

The *F*-statistic and *p*-value of two-way repeated measures ANOVA factors interaction (direction x environment/period) and main effects (direction and environment/period) in each paired-causality analyzing brain area. The corresponding main effects after Bonferroni's post hoc comparison with significant differences displayed in Fig. 4 were highlighted with the gray background.

Supplementary Table. 4: Statistical results of Fig. 5.

EPM				OFT			
Open arm entries				Inner zone entries			
Retrieval type/ Effect	Group x Time	Main effect of Group	Main effect of Time	Retrieval type/ Effect	Group x Time	Main effect of Group	Main effect of Time
Context retrieval	$F_{(1,13)}$=2.881, p=0.113	$F_{(1,13)}$=26.550, p<0.0001	$F_{(1,13)}$=5.827, p<0.05	Context retrieval	$F_{(1,13)}$=0.489, p=0.497	$F_{(1,13)}$=8.476, p<0.05	$F_{(1,13)}$=1.699, p=0.215
Cue retrieval	$F_{(1,13)}$=0.169, p=0.688	$F_{(1,13)}$=11.528, p<0.01	$F_{(1,13)}$=1.196, p=0.294	Cue retrieval	$F_{(1,13)}$=1.794, p=0.203	$F_{(1,13)}$=3.849, p=0.072	$F_{(1,13)}$=0.502, p=0.491
Time in open arm				Time in inner zone			
Retrieval type/ Effect	Group x Time	Main effect of Group	Main effect of Time	Retrieval type/ Effect	Group x Time	Main effect of Group	Main effect of Time
Context retrieval	$F_{(1,13)}$=2.261, p=0.157	$F_{(1,13)}$=8.318, p<0.05	$F_{(1,13)}$=2.193, p=0.162	Context retrieval	$F_{(1,13)}$=0.752, p=0.402	$F_{(1,13)}$=19.788, p<0.001	$F_{(1,13)}$=1.143, p=0.305
Cue retrieval	$F_{(1,13)}$=0.451, p=0.514	$F_{(1,13)}$=26.969, p<0.00001	$F_{(1,13)}$=0.586, p=0.458	Cue retrieval	$F_{(1,13)}$=0.476, p=0.503	$F_{(1,13)}$=22.947, p<0.0001	$F_{(1,13)}$=0.548, p=0.472

The *F*-statistic and *p*-value of two-way repeated measures ANOVA factors interaction (group x time) and main effects (group and time) in each memory retrieval type of behavioral tasks. The corresponding main effects after Bonferroni's post hoc comparison with significant differences displayed in Fig. 5 were highlighted with the gray background.

Supplementary Table. 5: Statistical results of Supplementary Fig. 2.

Context retrieval				Cue retrieval			
Power variation				Power variation			
Region/ Effect	Group x Time	Main effect of Group	Main effect of Time	Region/ Effect	Group x Time	Main effect of Group	Main effect of Time
PFC	$F_{(1,10)}$=0.786, p=0.469	$F_{(1,10)}$=3.982, p=0.074	$F_{(1,10)}$=3.427, p=0.053	PFC	$F_{(1,10)}$=9.891, p<0.001	$F_{(1,10)}$=3.686, p=0.084	$F_{(1,10)}$=48.291, p<0.0001
BLA	$F_{(1,10)}$=2.747, p=0.088	$F_{(1,10)}$=0.716, p=0.417	$F_{(1,10)}$=2.808, p=0.084	BLA	$F_{(1,10)}$=65.196, p<0.0001	$F_{(1,10)}$=7.102, p<0.05	$F_{(1,10)}$=12.451, p<0.0001
vHPC	$F_{(1,10)}$=7.792, p<0.05	$F_{(1,10)}$=0.555, p=0.474	$F_{(1,10)}$=3.184, p=0.063	vHPC	$F_{(1,10)}$=17.110, p<0.0001	$F_{(1,10)}$=0.132, p=0.724	$F_{(1,10)}$=4.130, p<0.05
Coherence variation				Coherence variation			
Region/ Effect	Group x Time	Main effect of Group	Main effect of Time	Region/ Effect	Group x Time	Main effect of Group	Main effect of Time
PFC-BLA	$F_{(1,10)}$=5.982, p<0.05	$F_{(1,10)}$=6.817, p<0.05	$F_{(1,10)}$=6.805, p<0.01	PFC-BLA	$F_{(1,10)}$=14.058, p<0.0001	$F_{(1,10)}$=26.399, p<0.0001	$F_{(1,10)}$=17.517, p<0.0001
PFC-vHPC	$F_{(1,10)}$=1.918, p=0.173	$F_{(1,10)}$=5.218, p<0.05	$F_{(1,10)}$=24.900, p<0.0001	PFC-vHPC	$F_{(1,10)}$=1.289, p=0.297	$F_{(1,10)}$=1.235, p=0.293	$F_{(1,10)}$=2.000, p=0.162
BLA-vHPC	$F_{(1,10)}$=17.542, p<0.0001	$F_{(1,10)}$=2.074, p=0.180	$F_{(1,10)}$=13.938, p<0.0001	BLA-vHPC	$F_{(1,10)}$=8.644, p<0.05	$F_{(1,10)}$=19.182, p<0.001	$F_{(1,10)}$=13.234, p<0.0001

The *F*-statistic and *p*-value of two-way repeated measures ANOVA factors interaction (group x time) and main effects (group and time) in each power intensity and paired-coherence observation brain area. The corresponding main effects after Bonferroni's post hoc comparison with significant differences displayed in Supplementary Fig. 2 were highlighted with the gray background.

Supplementary Table. 6: Statistical results of Supplementary Fig. 6.

EPM				OFT			
Open arm entries				Inner zone entries			
Retrieval type/ Effect	Group x Time	Main effect of Group	Main effect of Time	Retrieval type/ Effect	Group x Time	Main effect of Group	Main effect of Time
Context retrieval	$F_{(1,18)}$=3.329, p=0.063	$F_{(1,18)}$=8.342, p<0.01	$F_{(1,18)}$=0.857, p=0.367	Context retrieval	$F_{(1,18)}$=0.507, p=0.611	$F_{(1,18)}$=4.646, p<0.05	$F_{(1,18)}$=1.769, p=0.200
Cue retrieval	$F_{(1,18)}$=0.147, p=0.864	$F_{(1,18)}$=7.052, p<0.01	$F_{(1,18)}$=2.806, p=0.111	Cue retrieval	$F_{(1,18)}$=1.069, p=0.364	$F_{(1,18)}$=4.091, p<0.05	$F_{(1,18)}$=0.377, p=0.547
Time in open arm				Time in inner zone			
Retrieval type/ Effect	Group x Time	Main effect of Group	Main effect of Time	Retrieval type/ Effect	Group x Time	Main effect of Group	Main effect of Time
Context retrieval	$F_{(1,18)}$=1.024, p=0.379	$F_{(1,18)}$=2.639, p=0.099	$F_{(1,18)}$=3.008, p=0.100	Context retrieval	$F_{(1,18)}$=0.750, p=0.486	$F_{(1,18)}$=1.230, p=0.316	$F_{(1,18)}$=0.426, p=0.522
Cue retrieval	$F_{(1,18)}$=0.272, p=0.765	$F_{(1,18)}$=11.522, p<0.001	$F_{(1,18)}$=1.173, p=0.293	Cue retrieval	$F_{(1,18)}$=0.612, p=0.553	$F_{(1,18)}$=14.076, p<0.0001	$F_{(1,18)}$=2.013, p=0.173

The F-statistic and p-value of two-way repeated measures ANOVA factors interaction (group x time) and main effects (group and time) in each memory retrieval type of behavioral tasks. The corresponding main effects after Bonferroni's post hoc comparison with significant differences displayed in Supplementary Fig. 6 were highlighted with the gray background.
