# Supplementary material for: A prolonged stress rat model recapitulates some PTSD-like changes in sleep and neuronal connectivity
Source: Commun Biol. 2023 Jul 12;6:716. doi: 10.1038/s42003-023-05090-9 (PMC10338557; doi:10.1038/s42003-023-05090-9)
Supplement: Supplementary file 3 — Description of Additional Supplementary Files [file 42003_2023_5090_MOESM3_ESM.pdf]

Description of Additional Supplementary Files

File name: Supplementary Data 1

Description: The source data behind the main graphs in the paper.
