# Supplementary material for: A prolonged stress rat model recapitulates some PTSD-like changes in sleep and neuronal connectivity
Source: Commun Biol. 2023 Jul 12;6:716. doi: 10.1038/s42003-023-05090-9 (PMC10338557; doi:10.1038/s42003-023-05090-9)
Supplement: Supplementary file 5 — Reporting Summary [file 42003_2023_5090_MOESM5_ESM.pdf]

nature portfolio

Corresponding author(s): Fang-Chia Chang and Pei-Lu Yi

Last updated by author(s): Jun 15, 2023

Reporting Summary

Nature Portfolio wishes to improve the reproducibility of the work that we publish. This form provides structure for consistency and transparency in reporting. For further information on Nature Portfolio policies, see our Editorial Policies and the Editorial Policy Checklist.

Statistics

For all statistical analyses, confirm that the following items are present in the figure legend, table legend, main text, or Methods section.

n/a	Confirmed	
☐	☒	The exact sample size (n) for each experimental group/condition, given as a discrete number and unit of measurement
☐	☒	A statement on whether measurements were taken from distinct samples or whether the same sample was measured repeatedly
☐	☒	The statistical test(s) used AND whether they are one- or two-sided<br>*Only common tests should be described solely by name; describe more complex techniques in the Methods section.*
☐	☒	A description of all covariates tested
☐	☒	A description of any assumptions or corrections, such as tests of normality and adjustment for multiple comparisons
☐	☒	A full description of the statistical parameters including central tendency (e.g. means) or other basic estimates (e.g. regression coefficient) AND variation (e.g. standard deviation) or associated estimates of uncertainty (e.g. confidence intervals)
☐	☒	For null hypothesis testing, the test statistic (e.g. F, t, r) with confidence intervals, effect sizes, degrees of freedom and P value noted<br>*Give P values as exact values whenever suitable.*
☒	☐	For Bayesian analysis, information on the choice of priors and Markov chain Monte Carlo settings
☐	☒	For hierarchical and complex designs, identification of the appropriate level for tests and full reporting of outcomes
☒	☐	Estimates of effect sizes (e.g. Cohen's d, Pearson's r), indicating how they were calculated

Our web collection on statistics for biologists contains articles on many of the points above.

Software and code

Policy information about availability of computer code

Data collection	All the LFP signal was collected by OmniPlex System with PBX Preamplifier, DigiAmp A/D device, and OmniPlex Software (Plexon, TX, USA).<br>The behavioral tasks (EPM and OFT) were recorded by the software of EthoVision XT (Noldus, WUR, Netherlands).<br>The sleep-wake activity data in this study was collected by the software of ICELUS (M. R. Opp), written in LabView (National Instruments).

March 2021

Data analysis	All the LFP signals and the power spectrum of the sleep spindle recorded from the EEG raw data were analyzed by the Chronux tool package in MatLab (MathWorks, MA, USA). For processing LFP data, we used the function of mtspecgramc to estimate the power spectral in each recorded area, coherencyc function to calculate the cross-spectrum coherence within the circuitries, and Granger causality (G.C.) to analyze the bidirectional connectivities between collected areas. The mtspecgramc also examined the sleep spindle from Chronux.<br>The behavioral tasks (EPM and OFT) were analyzed by the software of EthoVision XT (Noldus, WUR, Netherlands).<br>The sleep-wake activity data in this study was analyzed by the software of ICELUS (M. R. Opp), written in LabView (National Instruments).<br>All the statistical data were analyzed by SPSS (IBM, New York, USA). The generalized estimating equations (GEEs) with exchangeable correlation structure and Bonferroni pairwise comparison were applied to estimate immobility behavior (including group x time factors) in both the main text and supplementary result owing to unequal data collections within groups. The first day of immobility behavior between control, SPS, and Day 1-MPS was analyzed via one-way ANOVA. The student's unpaired t-test compared the difference between the control and MPS groups in the corticosterone levels. The brain activity of LFP recording data (power intensity, coherence, and Granger causality (G.C.))was analyzed by two-way repeated measure analysis of variance (ANOVA) to include the factors of differences between group and pre-treated and post-treated fear memory retrieval factors. The student's unpaired t-test was used to compare the difference between the control and MPS groups in the theta power variation during behavior tasks. The two-way repeated ANOVA and Bonferroni pairwise post-hoc comparison were applied to analyze the group and time segment differences in behavior tasks (EPM and OFT) in the main text and supplementary result. The one-way ANOVA was used to assess the sleep-wake alterations, followed by Tukey's comparison post-hoc analysis in the main text and supplementary result. The α level of $p<0.05$ refers to significant differences between groups.

For manuscripts utilizing custom algorithms or software that are central to the research but not yet described in published literature, software must be made available to editors and reviewers. We strongly encourage code deposition in a community repository (e.g. GitHub). See the Nature Portfolio guidelines for submitting code & software for further information.

Data

Policy information about availability of data

All manuscripts must include a data availability statement. This statement should provide the following information, where applicable:

- Accession codes, unique identifiers, or web links for publicly available datasets
- A description of any restrictions on data availability
- For clinical datasets or third party data, please ensure that the statement adheres to our policy

The data that support the findings of this study in the main figures and tables are available from the corresponding author upon reasonable request. Supplementary information is available at communications biology's website.

Human research participants

Policy information about studies involving human research participants and Sex and Gender in Research.

Reporting on sex and gender	This study did not involve human subjects.
Population characteristics	This study did not involve human subjects.
Recruitment	This study did not involve human subjects.
Ethics oversight	This study did not involve human subjects.

Note that full information on the approval of the study protocol must also be provided in the manuscript.

Field-specific reporting

Please select the one below that is the best fit for your research. If you are not sure, read the appropriate sections before making your selection.

☒ Life sciences ☐ Behavioural & social sciences ☐ Ecological, evolutionary & environmental sciences

For a reference copy of the document with all sections, see nature.com/documents/nr-reporting-summary-flat.pdf

Life sciences study design

All studies must disclose on these points even when the disclosure is negative.

Sample size	The approved minimum sample size for the rodent's behavioral tasks and sleep-wake activity in the IACUC of NTU is six. In our study, we first evaluated whether the MPS protocol could effectively generate stressed behavior. Therefore, to prevent bias in behavioral experiments and enhance the trend of the results, we increased the sample size to nine in experiment 1 for both short-term (n=9) and long-term (n=9) measurements. And we also detected the sleep-wake activity during MPS protocol in the same group of rats (n=18) to evaluate the sleep alteration in experiment 3. As for experiment 2 and the sleep-wake activities during fear retrieval days in experiment 3, the sample size was six. We also applied SPS manipulations in two groups of rats (n=6 in each group) to compare the effects of behavioral alteration and sleep disruption between SPS and MPS models in the supplementary results. And the single-time retrieval MPS group rats (n=6) were compared with the repeated measure MPS group on immobility percentage difference. Each experiment's exact sample number and missing data number were reported in every figure legend for reference.

Data exclusions	We excluded the behavioral result of sleep during the fear memory retrieval tasks to prevent behavioral bias in the immobility percentage calculation. We also removed the LFP signals with artifact and noise during each time section of context and cue retrievals to prevent bias in the recording data analysis. The sleep-wake activity recording data with noise that affected the software analysis were also excluded. Details are described in the Methods.
Replication	For fear memory retrieval, behavioral tasks, and sleep-wake activity analysis results, we used at least two repeated trails to verify the same MPS protocol, and the reproduced results presented the same trend that all replication attempts were successful.
Randomization	The group allocation in this study was not randomized. We controlled the covariates in the experimental protocol by comparing the differences between treatment and control groups and comparing the pre-treated and post-treated effects in the same group.
Blinding	We were not blind to group allocation during data collection because the study did not include clinical data collection and was mainly conducted by one investigator. However, all the recorded videos were blindly provided to another experimenter to analyze the freezing behavior of rats, which can prevent the inter-individual variation of the immobility scoring.

Reporting for specific materials, systems and methods

We require information from authors about some types of materials, experimental systems and methods used in many studies. Here, indicate whether each material, system or method listed is relevant to your study. If you are not sure if a list item applies to your research, read the appropriate section before selecting a response.

Materials & experimental systems

n/a	Involved in the study
☐	☒ Antibodies
☒	☐ Eukaryotic cell lines
☒	☐ Palaeontology and archaeology
☐	☒ Animals and other organisms
☒	☐ Clinical data
☒	☐ Dual use research of concern

Methods

n/a	Involved in the study
☒	☐ ChIP-seq
☒	☐ Flow cytometry
☒	☐ MRI-based neuroimaging

Antibodies

Antibodies used	We used the corticosterone ELISA antibody (Enzo Life Sciences, Cat ADI-900-097, NY, USA) to detect the variation of serum corticosterone concentrations between MPS and control rats.
Validation	The corticosterone ELISA antibody is sensitive to detect the corticosterone levels in all species of animals with a high sensitivity of 27.0 pg/ml (range 32 - 20,000 pg/ml). More details are described on the manufacturer's website: https://www.enzolifesciences.com/ADI-900-097/corticosterone-elisa-kit/

Animals and other research organisms

Policy information about studies involving animals; ARRIVE guidelines recommended for reporting animal research, and Sex and Gender in Research

Laboratory animals	Male Sprague-Dawley rats (300-400g; BioLASCO, Taiwan)
Wild animals	This study did not involve wild animals.
Reporting on sex	This study only used male rats for experiments.
Field-collected samples	Each animal was hosted in a single 28x22x40 cm3 cage with the control of 12h:12h light:dark circadian rhythm, room temperature of 22.0 ± 2ºC, and humidity of 50 ± 5%. Food and drinking water were available ad libitum. The experiment protocol persisted for 50 days in this study.
Ethics oversight	All the experiments and health care were followed by the instructions of Institutional Animal Care and Use Committee (IACUC) of National Taiwan University (Approval number: NTU104-EL-00101).

Note that full information on the approval of the study protocol must also be provided in the manuscript.
